# Prevalence of *Sodalis glossinidius* and different trypanosome species in *Glossina palpalis palpali*s caught in the Fontem sleeping sickness focus of the southern Cameroon

**DOI:** 10.1051/parasite/2018044

**Published:** 2018-08-17

**Authors:** Sartrien Kanté Tagueu, Oumarou Farikou, Flobert Njiokou, Gustave Simo

**Affiliations:** 1 Molecular Parasitology and Entomology Unit, Department of Biochemistry, Faculty of Science, University of Dschang Dschang Cameroon; 2 Mission Spéciale d’Éradication des Glossines, Division Régionale Tsé-Tsé Adamaoua B.P. 263 Ngaoundéré Cameroon; 3 Department of Animal Biology and Physiology, Faculty of Science, University of Yaoundé I Yaoundé Cameroon

**Keywords:** *Glossina palpalis palpalis*, Symbiont, *Sodalis glossinidius*, *Trypanosoma* Sp, PCR

## Abstract

Tsetse flies are the cyclical vector of human and animal African trypanosomiasis. To improve vector control in order to achieve the elimination of human African trypanosomiasis (HAT) and boost the control of animal diseases, investigations have been undertaken on the tripartite association between tsetse, trypanosome, and symbionts. It is in this light that *Sodalis glossinidius* and different trypanosomes were identified in *Glossina palpalis palpalis* caught in Fontem in southern Cameroon. For this study, DNA was extracted from whole flies, and *S. glossinidius* and different trypanosome species were identified by polymerase chain reaction (PCR). Statistical analyses were performed to compare the trypanosome and *S. glossinidius* infection rates and to look for an association between these microorganisms. Of the 274 *G. p. palpalis* caught, 3.3% (9/274) were teneral. About 35% (96/274) of these flies harbored *S. glossinidius.* Of the 265 non-teneral flies, 37.7% were infected by trypanosomes. The infection rates of *Trypanosoma congolense “*forest type” and *Trypanosoma vivax* were 26.04% and 18.11%, respectively. About 6.41% of tsetse harbored mixed infections of *T. congolense* and *T. vivax*. Of the 69 tsetse with *T. congolense* infections, 33.33% (23/69) harbored *S. glossinidius* while 71.86% (69/96) of flies harboring *S. glossinidius* were not infected by trypanosomes. No association was observed between *S. glossinidius* and trypanosome infections. Some wild tsetse harbor *S. glossinidius* and trypanosomes, while others have no infection or are infected by only one of these microorganisms. We conclude that the presence of *S. glossinidius* does not favor trypanosome infections in *G. p. palpalis* of the Fontem focus.

## Abstract (fr)

La mouche tsé-tsé est le vecteur cyclique de la trypanosomiase humaine et de la plupart des trypanosomiases africaines animales. Pour améliorer la lutte antivectorielle afin d’éliminer la trypanosomiase humaine africaine et renforcer le contrôle des maladies animales, des études ont été entreprises sur l’association tripartite entre la mouche tsé-tsé, le trypanosome et les symbiotes. C’est dans cette optique que l’identification moléculaire de *Sodalis glossinidius* et de différentes espèces de trypanosomes a été réalisée chez *Glossina palpalis palpalis* capturé à Fontem dans le sud Cameroun. Pour cette étude, l’ADN a été extrait de la glossine entière et *S. glossinidius* et différentes espèces de trypanosomes ont été identifiés par PCR. Des analyses statistiques ont été réalisées pour comparer les taux d’infections des trypanosomes et de *S. glossinidius* et rechercher une association entre les deux microorganismes. Sur les 274 *G. p. palpalis* capturés, 3.3 % (9/274) étaient des mouches ténérales. Environ 35 % (96/274) de ces glossines abritaient *S. glossinidius*. Sur les 265 mouches non ténérales, 37.7 % étaient infectées par au moins une espèce de trypanosome. Les taux d’infection de *T. congolense* « type forêt » et *T. vivax* étaient respectivement de 26.04 % et 18.11 %. Près de 6.41 % des mouches tsé-tsé présentaient une infection mixte impliquant *T. congolense* « type forêt » et *T. vivax*. Sur les 69 glossines infectées par *Trypanosoma congolense*, 33.33 % hébergeaient *S. glossinidius* tandis que 71.86 % (69/96) des mouches hébergeant *S. glossinidius* n’avaient aucune infection trypanosomienne. Aucune association n’a été observée entre *S. glossinidius* et les infections trypanosomiennes. Certaines mouches tsé-tsé abritent *S. glossinidius* et des trypanosomes alors que d’autres n’ont aucune infection ou sont infectées par un seul de ces deux micro-organismes. Nous conclueons que dans le foyer de la maladie du sommeil de Fontem, la présence de *S. glossinidius* ne favorise pas les infections trypanosomiennes chez *G. p. palpalis*.

## Introduction

Tsetse flies are the cyclical vector of most trypanosome species that cause human and animal African trypanosomiasis. Two species of *Trypanosoma brucei* s.l. are responsible for human African trypanosomiasis (HAT): *Trypanosoma brucei gambiense* causes the chronic form of HAT in Western and Central Africa, while *Trypanosoma brucei rhodesiense* is responsible for the acute form of HAT in East Africa. The third subspecies, *Trypanosoma brucei brucei,* is not implicated in human infection but causes African animal trypanosomiasis (AAT), also called nagana. In addition to *Trypanosoma brucei brucei*, *Trypanosoma congolense, Trypanosoma vivax, Trypanosoma evansi* and *Trypanosoma simiae* also cause AAT. In Africa, the economic losses resulting from the negative impact of AAT on African agriculture are estimated to be higher than US$ 4.5 billion/year [1, 36]. Moreover, African farmers spend about 35 million dollars per year on trypanocidal drugs to protect and cure their cattle [5]. If African trypanosomiases were controlled, about 7 million km^2^ of tsetse infested area could be suitable for livestock and agriculture in Africa [30].

For HAT and AAT, very few drugs are available and resistance phenomena have been observed for some of them [3, 18]. To prevent trypanosome infections, no vaccine is available and several approaches have been investigated in order to improve vector control. It is in this light that in depth investigations targeting bacterial flora of tsetse flies have been undertaken in the last few decades. Indeed, tsetse flies harbor three symbionts including the obligate primary symbiont (essential) *Wigglesworthia glossinidia* [43], the secondary (non-essential) symbiont *Sodalis glossinidius* [7], and the third symbiont (non-essential) known as *Wolbachia* [33]. The secondary and facultative symbionts *S. glossinidius* [7] are enterobacteria, which are widely spread in numerous tissues of the fly [2]. They are suited as paratransgenic organisms due to their ability to survive in the same organs with trypanosomes [6]. *Sodalis glossinidius* affect host longevity and may influence the host’s ability to establish trypanosome infections [8]. Given their close association with their host’s biology and their large tissue tropism, *S. glossinidius* could be used to produce and deliver some specific molecules (antibodies) by expressing foreign genes designed to block pathogen development [10].

In some HAT foci of southern Cameroon, the presence of *S. glossinidius* has been reported to favor trypanosome infections [12]. However, the effect of *S. glossinidius* on trypanosome infections could depend on the trypanosome genotype [12, 15]. Moreover, the association between *S. glossinidius* and trypanosome infections could vary according to sampling areas, since the environmental conditions could affect the life history traits of tsetse flies as well as the association with their symbiotic microorganisms.

In this study, *S. glossinidius* and different trypanosome species were identified in *Glossina palpalis palpalis* caught in the Fontem sleeping sickness focus of the southern Cameroon, with the overarching goal of improving our understanding of the association between *S. glossinidius* and trypanosome infections.

## Methods

### Ethical statement

This study was carried out following the strict recommendations contained in the Guide for the Care and Use of Animals of the Department of Biochemistry of the University of Dschang.

### Study area

**Figure 1.**
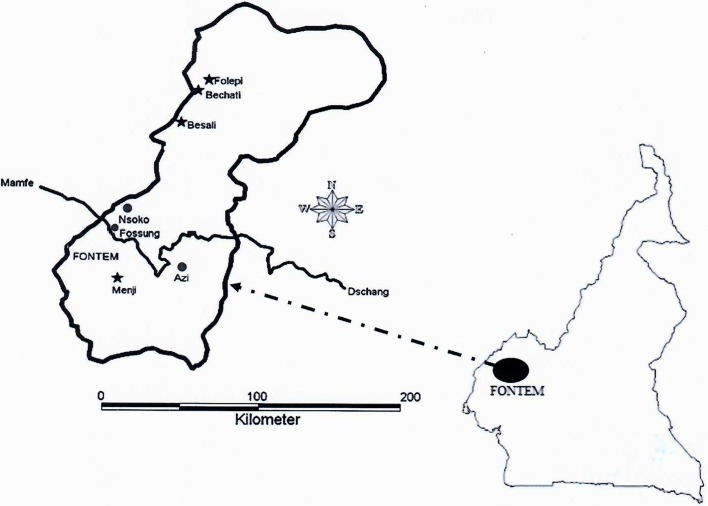
Map showing villages where entomological surveys where undertaken in the Fontem sleeping sickness focus. Stars: Villages where tsetse flies were trapped; circles: other villages. The road from Mamfé to Dschang is indicated in black.

The Fontem HAT focus (5°40′12 N, 9°55′33E) is located in the Lebialem division of the Southwest region of Cameroon. In this forest region, the climate is of the tropical moist type with a relief made up of hills and valleys. The region is crossed by many fast-moving streams. The main activity in the Fontem HAT focus is agriculture and breeding of small livestock and poultry. In addition to wild animals, several domestic animal species including dogs, pigs, sheep, and goats are found in this focus. For this study, the entomological surveys were performed in four villages of the Fontem HAT focus including Bechati, Folepi, Besali and Menji (Figure 1).

### Sampling of tsetse

Two entomological surveys were conducted in four villages (Figure 1) of the Fontem HAT focus using 30 traps in February 2015 and 12 traps in November 2016. In each of these villages, pyramidal traps [19] were set up for four consecutive days in various tsetse fly-favorable biotopes. The geographical coordinates of each trap were recorded with a global positioning system. In each village, tsetse flies were collected twice a day (from 9 to 10 am and from 3 to 4 pm). The collected flies were identified and numbered according to traps. Thereafter, the flies were sorted into teneral (young flies that had never taken a blood meal) and non-teneral flies. Each identified tsetse fly was subsequently put into a microtube containing ethanol at 95%. The microtubes were maintained at room temperature in the field. In the laboratory, they were stored at −20 °C until use.

### DNA extraction

DNA was extracted from whole tsetse fly using the cetyl trimethyl ammonium bromide (CTAB) method as described by Navajas *et al*. [29]. Briefly, the alcohol used to preserve each fly was evaporated by incubating the opened microtubes containing whole fly at 80 °C in an oven for about 1 h. Thereafter, each fly was disrupted with a pestle in CTAB buffer (CTAB 2%; 1 M Tris, pH 8; 0.5 M EDTA pH 8; 5 M NaCl). The disrupted tissues were incubated at 60 °C for 30 min before the addition of chloroform/isoamylic alcohol mixture (24/1; V/V). DNA was precipitated by addition of isopropanol (V/V) and a centrifugation at 13,000 rpm for 15 min. The DNA pellets were washed twice with 70% cool ethanol and then dried at room temperature. DNA pellets were finally re-suspended in 50 μL of sterile water before their storage at −20 °C until use.

### Detection of *S. glossinidius*

The presence of *S. glossinidius* was revealed by PCR with pSG2 direct (5′-TGAAGTTGGGAATGTCG-3′) and reverse (5′-AGTTGTAGCACAGCGTGTA-3’) primers as described by Darby *et al*. [9]. The PCR reactions were carried out in a DNA thermal cycler (Prime). Each amplification reaction was performed in a total volume of 25 μL containing 20 pmol of each primer, 2.5 μL of 10× reaction buffer, 2 mM of MgCl_2_, 200 mM of each dNTPs, 4 μL of DNA template, and 0.5 units of Taq DNA polymerase (New England Biolab 5 U/μL). The amplification reactions involved a denaturation step at 94 °C for 5 min followed by 40 amplification cycles made up of a denaturation step at 94 °C for 30 s, an annealing step at 56 °C for 30 s, and an extension step at 72 °C for 45 s. These amplifications were followed by a final extension step at 72 °C for 5 min. The amplified products were resolved by electrophoresis at 100 volts for 30 min on 2% agarose gel containing ethidium bromide. DNA bands were visualized under ultraviolet light.

### Detection of trypanosomes

Four sets of specific primers (Table 1) were used to identify *Trypanosoma brucei* s.l.*, Trypanosoma vivax,* and *Trypanosoma congolense* “forest” and “savannah” types. This identification was done by PCR as described by Herder *et al*. [16]. For this identification, each PCR reaction was carried out in a final volume of 15 μL containing 1.5 μL of 10× PCR reaction buffer, 1.5 mM of MgCl_2_, 200 mM of each dNTP, 10 picomoles of each primer (Table 1), 0.3 units of Taq DNA polymerase (New England Biolab 5 U/μL) and 3 μL of DNA extract. For each amplification reaction, a denaturation step at 94 °C for 5 min was followed by 40 amplification cycles. Each of these cycles included a denaturation step at 94 °C for 30 s, an annealing step for 30 s at 60 °C for *T. brucei* s.l.*, T. vivax,* and *T. congolense* “forest” and “savannah” types, and an extension step at 72 °C for 1 min. A final extension step was performed at 72 °C for 10 min. The amplified products were separated on 2% agarose gel containing ethidium bromide and visualized under UV illumination.

**Table 1.** Primers used for the identification of different trypanosome species.

Specificity	Primer sequence	References
*T. congolense* “forest type”	5′-GGACACGCCAGAAGGTACTT-3′ 5′-GTTCTCGCACCAAATCCAAC-3′	Masiga *et al*. [23]
*T. congolense* “savannah type”	5′-TCGAGCGAGAACGGGCACTTTGCGA-3′ 5′-ATTAGGGACAAACAAATCCCGCACA-3′	Moser *et al*. [27]
*T. brucei* s.l.	5′-CGAATGAATATTAAACAATGCGCAG-3′ 5′-AGAACCATTTATTAGCTTTGTTGC-3′	Masiga *et al*. [23]
*T. vivax*	5′-CTGAGTGCTCCATGTCCCAC-3′ 5′-CCACCAGAACACCAACCTGA-3′	Masiga *et al*. [23]

### Statistical analysis

The statistical analyses were performed using StataCorp 2015 statistical software, release 14 (StataCorp LP; College Station, TX, USA). Chi-squared tests were used to compare the infection rates of *S. glossinidius* and different trypanosome species between villages. The differences were considered significant when the *p*-values were lower than 0.05. To see whether the presence of *S. glossinidius* could favor trypanosome infections, a generalized linear model using StataCorp 2015 software with 95% confidence intervals (CIs) was used. For these analyses, *T. vivax* was excluded because its lifecycle is exclusively completed within the mouthparts of the tsetse fly.

## Results

### Entomological surveys

During the two entomological surveys, 274 tsetse flies were collected. Details regarding results of entomological surveys are reported in Table 2. Of the 274 tsetse flies collected, 9 (3.28%) teneral flies were identified (Table 2). No teneral flies were identified at Besali. The mean apparent fly density per trap per day (ADT) varied from 0.2 to 2.79, with an average of 1.63. The highest ADT was recorded at Folepi.

**Table 2.** Results of entomological surveys and infection rates of *S. glossinidius* according to villages

Survey villages	Number of traps	Number of flies captured	ADT	Number teneral flies (%)	Number of flies analyzed	Number of flies hosting *S. glossinidius* (%)	95% CI
Bechati	12	54	1.12	1 (1.85)	54	21 (38.89)	27.45 – 53.22
Besali	5	4	0.2	0 (0.00)	4	1 (25.00)	3.35 – 76.22
Folepi	13	145	2.79	7 (4.83)	145	46 (31.72)	23.99 – 39.35
Menji	12	71	1.48	1 (1.41)	71	28 (39.44)	27.96 – 50.4
Total	42	274	1.63	9 (3.28)	274	96 (35.04)	
*p*-value						0.5801	

ADT: apparent density per trap per day; (%): *S. glossinidius* infection rate; CI: Confidence interval.

### Molecular identification of *S. glossinidius*

Of the 274 flies analyzed, 96 were positive for *S. glossinidius,* yielding an overall infection rate of 35.04%. The highest infection rate of 39.44% [95% CI = 27.96% – 50.4%] was observed at Menji, and the lowest rate of 25% [95% CI = 3.35% – 76.22%] at Besali. Despite the variations observed in the infection rates, no significant difference (*p-*value: 0.5801) was observed between villages (Table 2).

### Molecular detection of different trypanosome species

Of the 265 non-teneral tsetse flies analyzed, 100 (37.73%) were infected by at least one trypanosome species. Of these 100 infected flies, 69 (26.04%) were infected due to *T. congolense* “forest type”, 48 (18.11%) to *T. vivax,* and 17 (6.41%) were mixed infections (Table 3). No infections due to *T. brucei* s.l. were observed. The highest infection rates of 40% [95% CI = 29.24% – 51.82%] for *T. congolense* and 25% [95% CI = 3.35% – 76.22%] for *T. vivax* were observed at Menji and Besali, respectively. The lowest infection rates of 16.98% [95% CI = 9.08% – 29.53%] for *T. congolense* and 15.94% [95% CI = 10.73% – 23.03%] for *T. vivax* were observed at Bechati and Folepi, respectively. For *T. congolense* “forest type”, a significant difference was found between villages. For *T. vivax*, no significant difference was found between villages. Of the 100 tsetse flies with trypanosome infections, 17 (17%) were co-infected by *T. vivax* and *T. congolense* “forest type”, yielding an overall co-infection rate of 6.41% (17/265). Between villages, significant differences (*p*-value: 0.0454) were observed in the co-infection rates (Table 3).

**Table 3.** Trypanosome infections according to villages.

Villages	No of flies captured	No of flies analyzed	T+	Tcf (%)	95% CI	Tv (%)	95% CI	Tcf/Tv (%)	95% CI
Bechati	54	53	18^a^ (33.96)	9 (16.98)	9.08 – 29.53	11 (20.75)	11.88 – 33.72	2 (3.77)	0.94 – 13.87
Besali	4	4	1^a^ (25.00)	1 (25.00)	3.35 – 76.22	1 (25.00)	3.35 – 76.22	1 (25.00)	3.35 – 76.22
Folepi	145	138	48^a^ (34.78)	31 (22.46)	16.26 – 30.17	22 (15.94)	10.73 – 23.03	5 (3.62)	1.52 – 8.41
Menji	71	70	33^a^ (47.14)	28 (40.00)	29.24 – 51.82	14 (20.00)	12.22 – 30.99	9 (12.86)	6.83 – 22.90
Total	274	265	100^a^ (37.73)	69 (26.04)		48 (18.11)		17 (6.41)	
*p*-value				0.0195		0.8073		0.0454	

No: number; (%): trypanosome infection rate; T+: tsetse flies with trypanosome infections; Tcf: *Trypanosoma congolense* “forest type”; Tv: *Trypanosoma vivax*; Tcf/Tv: co-infection of *Trypanosoma congolense* “forest type” and *Trypanosoma vivax*. CI: Confidence interval.

a some of these tsetse flies were co-infected by different trypanosome species;

### Co-infection of trypanosomes and *S. glossinidius*

Of the 265 tsetse flies that were simultaneously analyzed for the presence of trypanosomes and *S. glossinidius*, 92 (34.72%) harbored *S. glossinidius* and 100 (37.73%) were infected by at least one trypanosome species. The number of tsetse flies with trypanosome infections is higher than the number of flies with *S. glossinidius*. Considering the fact that *T. vivax* is found exclusively in the mouthparts, the 21 tsetse flies with only *T. vivax* infections were excluded from the analyses performed here. As a consequence, only 69 flies with *T. congolense* “forest type” were considered during investigations on the association between *S. glossinidius* and trypanosome infections. Of the 69 flies infected by *T. congolense* “forest type”, 23 (33.33%) also harbored *S. glossinidius* (S + Tcf +), while the remaining 46 (66.67%) were without *S. glossinidius* (S-Tcf +) (Table 4). About 71.86% (69/96) of flies harboring *S. glossinidius* were not infected by trypanosomes. The analyses performed to see if the presence of *S. glossinidius* could have an impact on the trypanosome infections (Tcf +) revealed no significant association (*r* = −0.0831; *p* = 0.7785; [95% CI = −0.66 – 0.5]) between these two micro-organisms (Table 4).

**Table 4.** *Sodalis glossinidius* and *T. congolense* co-infections according to villages.

Villages	Number of flies analyzed	S+Tcf−	S+Tcf+	S-Tcf+	S-Tcf−	Flies positive to *S. glossinidius*	Flies infected by Tcf
Bechati	53	19	2	7	25	21	9
Besali	4	0	1	0	3	1	1
Folepi	138	33	10	21	74	43	31
Menji	70	17	10	18	25	27	28
Total	265	69	23	46	127	92	69
*r*	−0.0831						
*p*-value	0.7785						
95% CI	[−0.66 – 0.5]						

CI: Confidence interval; *r*: generalize linear model coefficient; S+Tcf+: tsetse flies co-infected by *S. glossinidius* and *Trypanosoma congolense* “forest type”; S+Tcf−: tsetse flies with *S. glossinidius* and without *Trypanosoma congolense* “forest type” infection; S-Tcf+: tsetse flies with *Trypanosoma congolense* “forest type” and without *S. glossinidius*; S-Tcf−: tsetse flies without *S. glossinidius* and *Trypanosoma congolense* “forest type”.

## Discussion

We carried out several studies on tsetse flies to understand their biology and their bacterial flora, and also to identify the parasites infecting these flies. *S. glossinidius* and different trypanosome species were investigated in tsetse flies caught in the Fontem HAT focus of Cameroon, with the overarching goal of improving our knowledge on the vector competence of tsetse flies. Results of entomological surveys confirm *G. p. palpalis* as the only tsetse species in this focus. They are in agreement with previous observations [26, 32], highlighting the role of *G. p. palpalis* in the transmission of African trypanosomiases in the Fontem HAT focus. The apparent density of the tsetse per trap per day (ADT) of 1.63 is very low when compared to 7.9 and 4.85 obtained 20 (1998) and 10 (2007) years ago in the same area by Morlais *et al*. [26] and Njitchouang *et al*. [32]. This decrease of ADT could be linked to local disturbance resulting more likely from bush clearing and population growth which has induced climatic and environmental modifications that affected tsetse biotopes. These modifications occurred with time and subsequently, have induced some changes in the composition and host availability, the nutritional behavior of tsetse, and the transmission dynamics of trypanosomes [28, 38, 41].

The presence of *S. glossinidius* in *G. p. palpalis* caught in Cameroon confirms results obtained in two other HAT foci of the forest regions of southern Cameroon [12]. The *S. glossinidius* infection rate of 35.04% obtained here is lower than the 64.4% and 45.3% reported in *G. p. palpalis* caught at the Bipindi and Campo HAT foci of Cameroon, which are located more 400 km from the Fontem HAT focus [12]. This is higher than the 9.3% reported in Liberia for the same tsetse species [24]. These differences could be linked to sampling areas since each area is characterized by specific environmental factors that affect tsetse biology as well as the symbiotic association, and subsequently the vertical transmission of *S. glossinidius* from mother to offspring. When environmental factors are stable, like in insectariums, the transmission rate of symbiotic micro-organisms from mother to offspring is quite high [37]. For colonies of *G. p. gambiense* and *G. m. morsitans* from Burkina Faso that were maintained in insectariums, the infection rates of *S. glossinidius* reached 100% [14].

Comparing the *S. glossinidius* infection rates between different tsetse species [20, 24, 42], the high variation observed could be explained by the intrinsic characters of each tsetse species. For the same stimuli (internal or external), tsetse species will respond differently (differential behaviors) because of their specific biological characters that induce variations in the molecular interactions between tsetse and its symbiotic microorganisms and consequently, in the infection rates of different symbionts. The high variation reported above could also result from certain differences in analytical methods. In our study for instance, whole tsetse fly was used while in previous studies, tsetse flies were dissected and investigations were performed on isolated tissues.

The identification of *T. congolense* and *T. vivax* confirms results obtained in the same area [26, 40]. In the same villages, these trypanosomes have previously been detected in tsetse and different domestic animals; indicating their active transmission. Our results corroborate those reported in western, eastern and central African where the same trypanosome species were detected in different tsetse species [11–13, 23, 26, 34, 35]. They are also in agreement with results obtained in a variety of wild and domestic animals despite the fact that none of these animals were investigated in this study [17, 31, 39, 40]. This wide distribution of *T. vivax* and *T. congolense* indicates their ubiquity and the presence of appropriate vertebrate hosts. The high infection rate of *T. congolense* forest “type” is linked to the geographical localization of the Fontem HAT focus because this species is mainly found in the forest regions. It can also be explained by the fact that whole tsetse fly were analyzed, and the infection rates reported here are the combination of infections occurring in different tissues such as mouthparts and midguts.

Although single infections were predominant, about 6.41% mixed infections involving *T. congolense* forest and *T. vivax* were identified. This result corroborates data reported in tsetse [25, 31] and domestic animals [39] of Cameroon and other African countries [21, 22, 35]. It is important to point out that the identification of different trypanosome species was performed on whole tsetse fly and consequently, we do not know whether these infections were immature or mature, and which organ or tissue was infected. It is also unknown whether the mixed infections identified were from the same organ or tissue. Without such information, it becomes difficult to foresee the impact of mixed infections on the transmission and the dynamics of trypanosomes from tsetse to vertebrate hosts. Remarkably, the trypanosomes (*T. vivax* and *T. congolense*) reported in mixed infections can be found in their metacyclic forms in the mouthparts of tsetse flies. In such conditions, these trypanosomes can be simultaneously transmitted to vertebrate hosts. The mixed infections identified in this study therefore highlight a high probability that tsetse flies harbor or transmit several trypanosome species. If such transmission occurs, it becomes important to know which parasite could develop rapidly and what could be the impact of such infections on the transmission dynamics and animal health. Investigations on mixed infections in vertebrate hosts have shown mutual suppression and their advantages for the infected host [4]. As for vertebrate hosts, understanding how mixed infections evolve in tsetse flies and their potential impacts on the transmission dynamics of trypanosomes are areas for future investigation. The high infection rate observed in this study indicates active transmission of different trypanosome species. It highlights that the animal African trypanosomiases remain a serious threat to animal health and to the rural economy in the villages of the Fontem HAT focus.

The identification of tsetse flies with co-infections of *S. glossinidius* and trypanosomes, and other with trypanosome infections and without *S. glossinidius,* or with *S. glossinidius* and no trypanosome infection, corroborates results obtained elsewhere [12]. These results show that different scenarios in the tripartite association between tsetse, trypanosomes and symbiotic microorganisms can occur in natural populations of tsetse flies. In addition to these scenarios, the teneral or non-teneral status of tsetse fly and the first blood meal taken on non-infected vertebrate host could also affect the ability of tsetse to become infected and therefore, could negate any positive influence that *S. glossinidius* might have on tsetse susceptibility.

Our results showing no significant association (*r* = −0.083; *p* = 0.778) between the presence of *S. glossinidius* and trypanosomes infections indicate that the presence of *S. glossinidius* seems not absolutely necessary for trypanosome infections in the Fontem HAT focus. These findings are in line with those reported in other tsetse species like *G. austeni* [42], *G. brevipalpis, G. morsitans morsitans* and *G. pallidipes* [11] where no significant association has been reported between the presence of *S. glossinidius* and trypanosome infections. These results contrast with previous ones where the presence of *S. glossinidius* was reported to favor trypanosome infections not only in *G. p. palpalis* of other HAT foci of Cameroon [12], but also in other tsetse species [42]. These results highlight differences in the tripartite association between tsetse fly, *S. glossinidius* and trypanosomes. As a result of tsetse biology and environmental factors impacting the association between tsetse fly and its symbiotic micro-organisms, the tripartite association between tsetse, *S. glossinidius* and trypanosomes seems to vary according to tsetse species, and also to different populations (from different tsetse infested areas) of the same tsetse species. To better understand this tripartite association, more in-depth investigations on natural populations of different tsetse species in various tsetse infested areas are becoming important.

Our study on the tripartite association was based on presence/absence of trypanosome or *S. glossinidius*. Instead of focusing on this presence/absence, the genetic characterization of *S. glossinidius* strains could add additional value. In fact, Geiger *et al*. [15] have demonstrated that the tripartite association could be affected by specific genotypes of *S. glossinidius* and some trypanosome species, such as *T. b. gambiense* and *T. b. brucei*. Some specific *S. glossinidius* genotypes could affect the vectorial competence of *G. p. gambiensis* and *G. m. morsitans* for some trypanosome species [14]. The genetic characterization of bacteria populations could therefore enable us to improve our understanding of the tripartite association, and to better understand the real contribution of *S. glossinidius* to this association.

## Conclusion

This study has shown that, within the same tsetse infested area, the infection rates of *S*. *glossinidius* and different trypanosome species do not vary significantly between villages. Our results also show that in natural conditions, some tsetse flies simultaneously harbor *S. glossinidius* and trypanosomes, while others have no infection or can be infected by only one of these microorganisms. They do not confirm previous results reporting that the presence of *S. glossinidius* seems to favor trypanosome infections in *G. p. palpalis*. The relationship between *S. glossinidius* and trypanosome infections seems to vary according to the tsetse infested areas. Genetic comparisons between *S. glossinidius* populations found in tsetse flies co-infected with both *S. glossinidius* and trypanosomes, and those found in tsetse flies without trypanosome infections, could enable us to deepen our understanding of the role of *S. glossinidius* in the vector competence of *G. p. palpalis.*
